# Discrimination of Anti-Donor Response in Allogeneic Transplantation Using an Alloreactive T-Cell Detection Assay

**DOI:** 10.3389/ti.2025.13879

**Published:** 2025-01-28

**Authors:** Ryosuke Arata, Naoki Tanimine, Akhmet Seidakhmetov, Kentaro Ide, Yuka Tanaka, Hideki Ohdan

**Affiliations:** Department of Gastroenterological and Transplant Surgery, Graduate School of Biomedical and Health Sciences, Hiroshima University, Hiroshima, Japan

**Keywords:** immune monitoring, allogeneic transplantation, alloreactive T-cell, rejection, tolerance

## Abstract

Understanding donor-reactive T-cell behavior post-transplantation is challenging owing to the rarity and diversity of these cells. Here, we aimed to evaluate the relevance of an assay for rapidly detecting alloreactive T cells in a mouse transplantation model. After 18 h of one-way mixed lymphocyte reaction (MLR) culture with pre-activated donor-derived stimulators, CD4^+^ and CD8^+^ donor-reactive T cells were identified by CD154 and CD137 expression, respectively. Using full MHC mismatched mouse skin transplant models, we observed an increased donor-reactive T-cell proportion by direct presentation with elevated interferon gamma and granzyme B production 7 days post-transplantation, before graft rejection. Immunosuppression with CTLA-4 IgG and anti-CD154 antibody varied depending on donor-recipient strain combinations. On day 7, donor-reactive CD8^+^ T-cell proportions were lower in the tolerance model (BALB/c to C3H/HeJ) than in the rejection model (BALB/c to C57BL/6); conventional proliferation readout after 4 days of MLR could not distinguish these responses. Overall, although the conventional readout for evaluating T-cell proliferation following an MLR quantifies the precursor frequency of alloreactive T cells, the assay reported herein assesses T-cell activation markers after a short-term MLR to characterize immediate immune status. These findings offer a promising tool to elucidate immune responses post-transplantation.

## Graphical Abstract

**Figure:**
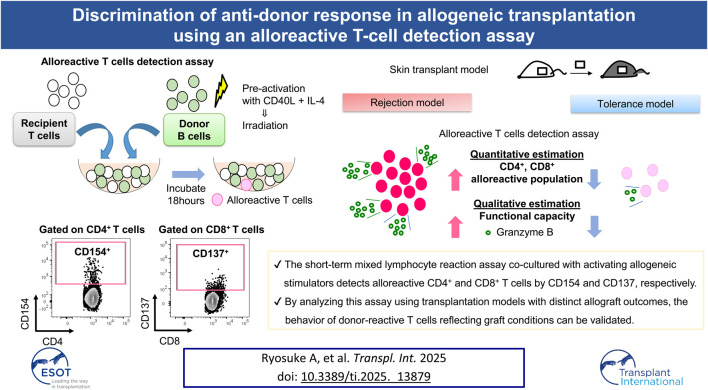

## Introduction

T cells play pivotal roles in orchestrating immune responses after solid organ transplantation [1]. Through their unique T-cell receptors (TCRs), these cells recognize antigens presented on the peptide-major histocompatibility complex (pMHC) on antigen-presenting cells (APCs) [2]. After transplantation, alloreactive T cells can enhance and mediate immune responses, resulting in organ damage and memory formation [3]. Donor-reactive T cells, which are quantitatively rare, reflect the anti-donor immune status, which may elucidate the hidden mechanisms underlying complex interactions in T-cell activation and regulation during the immune response [4]. Next-generation sequencing is a robust tool for comprehensive and high-throughput TCR profiling and facilitates the determination of the entire T-cell repertoire profile and tracing of antigen-specific T cells [5]. Although the MHC multimer is also an excellent marker for detecting antigen-specific T-cell clones in the total pool [6], it is challenging to identify alloreactive T cells in the clinical context owing to alloantigen diversity and variability [7].

Mixed lymphocyte reaction (MLR) is a classical and reliable method for estimating T-cell response in allogeneic transplantation and is useful for detecting clones against heterogenous allo-antigens. Previously, a novel comprehensive alloreactive T-cell detection (cATD) assay was developed using the MLR platform with activating markers (CD137 and CD154) [8]. In the present study, we aimed to investigate the relevance of alloreactive T cells via a direct pathway detected using this assay in a transplantation model. Specifically, we monitored alloreactive T cells in a mouse skin transplant model to clarify the importance of detected alloreactive T cells for rejection. In addition, we investigated whether this method could be useful to estimate the immune tolerance status.

## Materials and Methods

### Flow Cytometry

The following antibodies were used: anti-AF700-CD8a (53-6.7), anti-APC-CD154 (MR1), anti-APCCy7-CD8a (53-6.7), anti-PE-CD137 (17B5), anti-PE-CD4 (GK1.5), anti-PerCPcy5.5-CD3 (17A2), anti-BV421-CD62L (MEL-14), anti-BV421-granzyme B (GZMB; QA18A28), anti-BV605-CD4 (RM4-5), anti-BV711-CD44 (IM7), and anti-BV711-interferon gamma (IFN-γ; XMG1.2), purchased from BioLegend (San Diego, CA, United States). Anti-APCCy7-CD19 (1D3) and anti-PE Cy7-FoxP3 (FJK-16s) were purchased from BD Biosciences (San Jose, CA, United States). Nonspecific FcγR binding of labeled monoclonal antibodies (mAbs) was blocked using anti-mouse CD16/32 (2.4G2; BD Pharmingen, Hamburg, Germany). Dead cells were excluded from analysis using the forward Zombie Aqua Fixable Viability Kit (BioLegend), the Zombie NIR Fixable Viability Kit (BioLegend), or 7-aminoactinomycin D (7-AAD; BD Biosciences) staining. For intracellular staining, cells were fixed and permeabilized using the FoxP3/Transcription Factor Staining Buffer Set (BD Biosciences), according to the manufacturer’s instructions. To assess cytokine production, the cells were stimulated using monensin (BD Biosciences) in a culture medium at 37°C in a 5% CO_2_ incubator for 4 h prior to staining. The data were collected using LSRFortessa X-20, FACS Canto II, or FACS Celesta (BD Biosciences) and were analyzed using FlowJo v. 10 (Tree Star, Ashland, OR, United States).

### Mice

C57BL/6 (H-2Db), BALB/c (H-2Dd), and C3H/HeJ (H-2Dk) mice were purchased from CLEA (Osaka, Japan) and maintained in a pathogen-free animal facility of Hiroshima University, Hiroshima, Japan. Female mouse were used at an age of 10–12 weeks. When indicated, the mice were euthanized through cervical dislocation after isoflurane inhalation. All efforts were made to minimize animal suffering [9]. This study was performed in strict accordance with the “Guide for the Care and Use of Laboratory Animals” prepared by the Institute of Laboratory Animal Resources and published by the National Institutes of Health. All mice received humane care in compliance with the Principles of Laboratory Animal Care formulated by the National Society for Medical. The experimental protocol was approved by the Ethics Review Committee for Animal Experimentation of the Graduate School of Biomedical Sciences, Hiroshima University (Permit Number: A23-17). A part of this work was performed at the Research Facilities for Laboratory Animal Science, Natural Science Center for Basic Research and Development (N-BARD), Hiroshima University.

### Skin Transplantation

Full-thickness skin grafts were transplanted onto the left lateral dorsum of a recipient. Briefly, donor skin tissues were removed from the tails and trimmed into 10 mm × 10 mm strips. Recipient mice were anesthetized using intraperitoneal injection of xylazine (5 mg/kg body weight) and ketamine (100 mg/kg body weight). Skin tissues of the same size were removed from the recipients’ backs and replaced with donor grafts. The skin grafts were covered with bandages for 5 days, and graft survival was evaluated through daily visual inspection. Rejection was defined as destruction of >95% of the skin transplant [10]. An MHC full-mismatch BALB/c into C57BL/6 combination was employed as a rejection model. A BALB/c into C57BL/6 or C3H/HeJ combination previously reported as a tolerance induction model treated with CTLA-4 IgG (abatacept, 200 μg; Bristol-Myers Squibb, Braine-l’Alleud, Belgium) on days 0, 2, 4, and 6, and anti-CD154 antibody (MR1, 250 μg; BioLegend, San Diego, CA, United States) on days 0, 2, and 4 [11] was used for monitoring peripheral tolerance induction.

### cATD Assay

We prepared mononuclear cell suspensions of BALB/c mouse spleens and purified the B cells via positive selection using CD19 MicroBeads (Miltenyi Biotec, San Diego, CA, United States) in an autoMACS Pro Separator (Miltenyi Biotec), according to the manufacturer’s instructions [9]. The purity of the sorted cells was consistently >95%. Using a cocktail of recombinant mouse CD40L multimer (100 ng/mL; AdipoGen, San Diego, CA, United States) and recombinant mouse IL-4 (10 ng/mL; R&D Systems, Minneapolis, MN, United States), activated B cells were generated by culturing 0.2 × 10^6^ cells/mL at 37°C under 5% CO_2_ for 24 h. All cell cultures were performed in complete medium [RPMI 1640 medium (Nacalai Tesque, Kyoto, Japan) supplemented with 5% fetal bovine serum (SERANA, Pessin, Germany), 100 mM sodium pyruvate (Thermo Fisher Scientific, Waltham, MA), 100 U/mL penicillin–streptomycin (Thermo Fisher Scientific), 1% HEPES buffer (Thermo Fisher Scientific), and 50 µM 2-ME] in a 48-well flat-bottom plate. Using activated B cells as stimulators, MLR culture was performed, after which alloreactive T cells were identified. Prior to culturing, the stimulators were irradiated with 40 Gy. Responder T cells were purified from recipient splenocytes via negative selection, using a Pan T-Cell isolation kit (Miltenyi Biotec) in the autoMACS Pro Separator (Miltenyi Biotec), according to the manufacturer’s instructions. The purity of the sorted cells was consistently >95%. Responders and stimulators were co-cultured at a 1:1 ratio (10^6^ cells each) in 96-well U-bottom plates, with 200 µL complete medium containing APC-conjugated anti-CD154-labeled mAbs (MR1, 1 μL; BioLegend) for 18 h. Protein transport inhibitor (monensin, 2 μL; BD Biosciences) was added to the culture medium for the last 4 h of incubation. Alloreactive CD4^+^ and CD8^+^ T cells were identified as CD3^+^CD4^+^CD154^+^ and CD3^+^ CD8^+^CD137^+^ responders, respectively. We collected at least 100,000 counts during flow cytometry acquisition for detecting 0.1% population to keep the coefficient of validation up to 10%.

### Proliferation Assay

Recipient splenocytes were labeled with 5 µM carboxy fluorescein succinimidyl ester (CFSE; Molecular Probes) for 5 min prior to culturing. The activated B-cell stimulators were prepared as described in *cATD Assay*. Responders and stimulators were co-cultured at a 1:1 ratio (2 × 10^5^ cells each) for 4 days in 96-well U-bottom plates with 200 µL medium. Attenuation of CFSE fluorescence intensity was evaluated as proliferating activity gated on CD4^+^ and CD8^+^ T cells. Mitotic index (MI) was calculated as previously described [12, 13].

### Statistical Analysis

Statistical analyses were performed using JMP 16 (SAS Institute, Cary, NC, United States). Chi-square or Fisher’s exact test was used to compare categorical variables, and Student’s *t*-test or Mann–Whitney U-test was used for continuous variables. Comparisons between groups were made using the one-way analysis of variance (ANOVA), and significant differences were examined using Tukey–Kramer’s multiple-comparison post-hoc test. Differences with p < 0.05 were considered statistically significant.

## Results

### cATD Assay Detected Sensitization Leading to Acute Rejection in the Mouse Skin Transplantation Model

**FIGURE 1:**
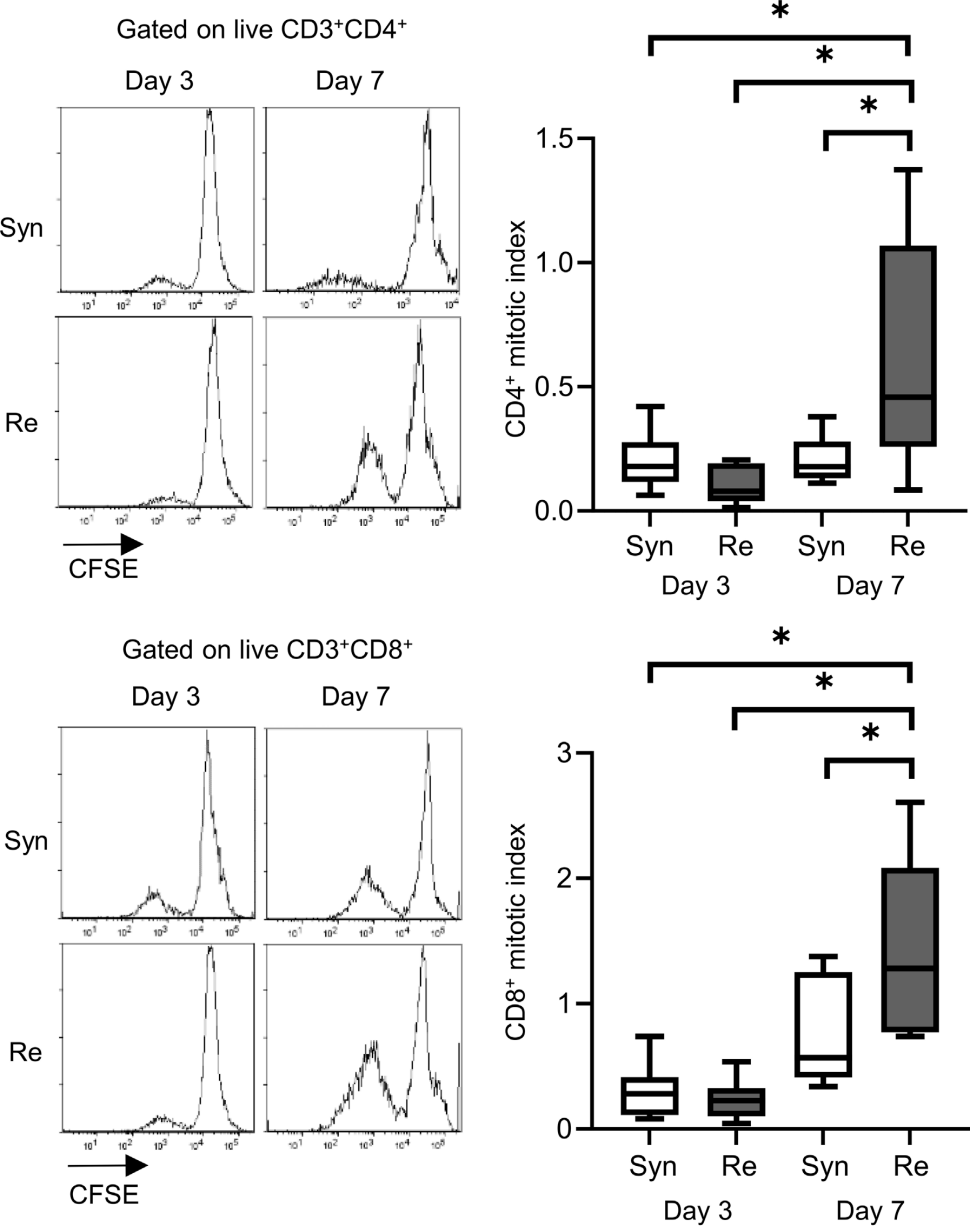
Proliferation assay after mouse skin transplantation. The representative flow plots and box-and-whisker plots of the mitotic index show the proliferation capacity of CD4^+^ (upper) and CD8^+^ (lower) T cells from recipients of the syngeneic model (Syn, C57BL/6 into C57BL/6) and rejection model (Re, BALB/c into C57BL/6) at 3 and 7 days after transplantation. *p < 0.05. The data were generated from four independent experiments (n = 6). One-way ANOVA and Tukey’s multiple-comparison test were employed for statistical analysis.

**FIGURE 2:**
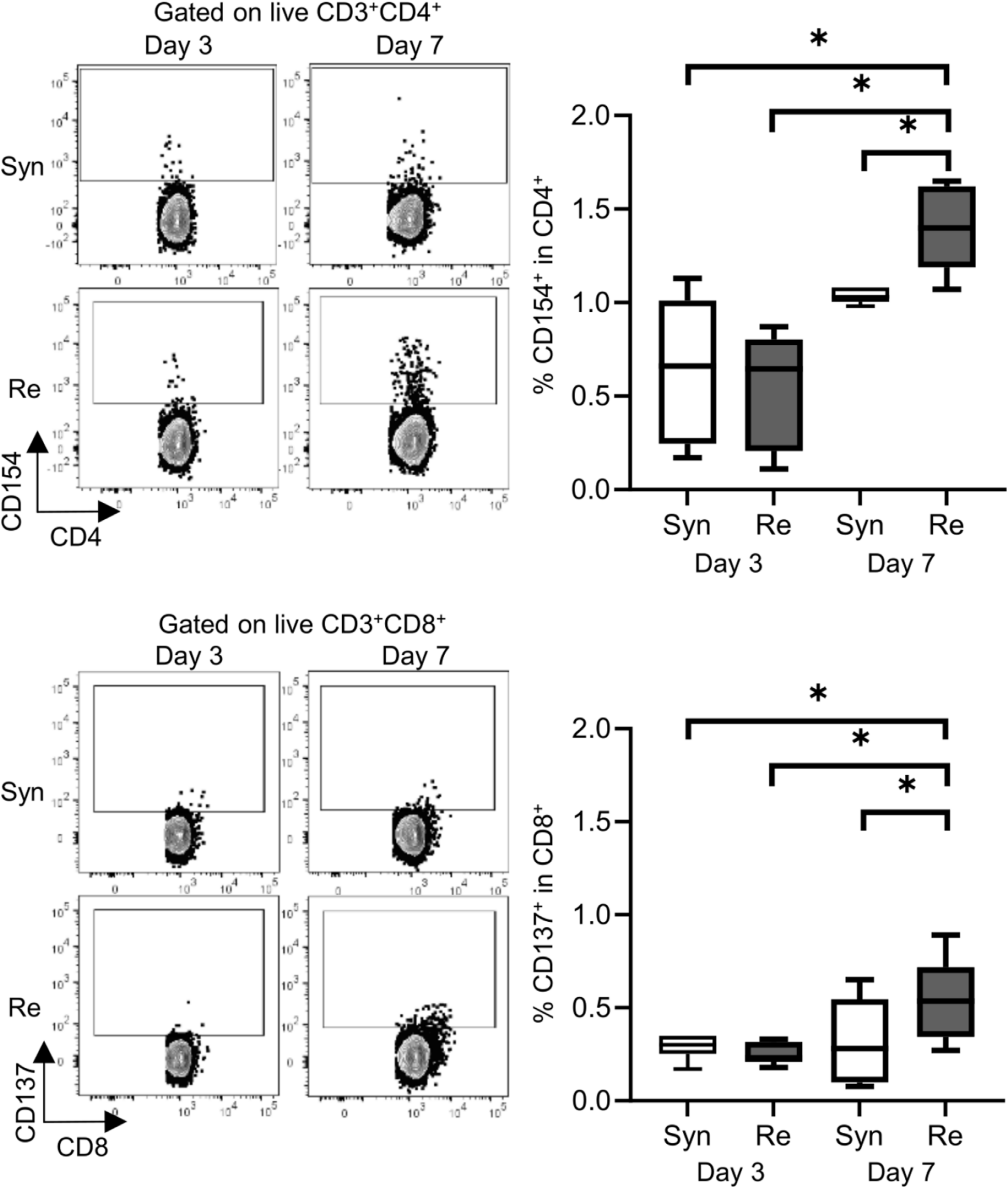
Alloreactive T-cell detection assay revealed donor-reactive T cells in the mouse skin transplantation model. The representative flow plots show the alloreactive population defined by CD154^+^ in CD4^+^ T cells (upper) and CD137^+^ in CD8^+^ T cells (lower) from recipients of the syngeneic model (Syn, C57BL/6 into C57BL/6) and rejection model (Re, BALB/c into C57BL/6). The box-and-whisker plots show the proportion of donor-reactive T cells at 3 and 7 days after transplantation. *p < 0.05. The data were generated from four independent experiments (n = 6). One-way ANOVA and Tukey’s multiple-comparison test were employed for statistical analysis.

**FIGURE 3:**
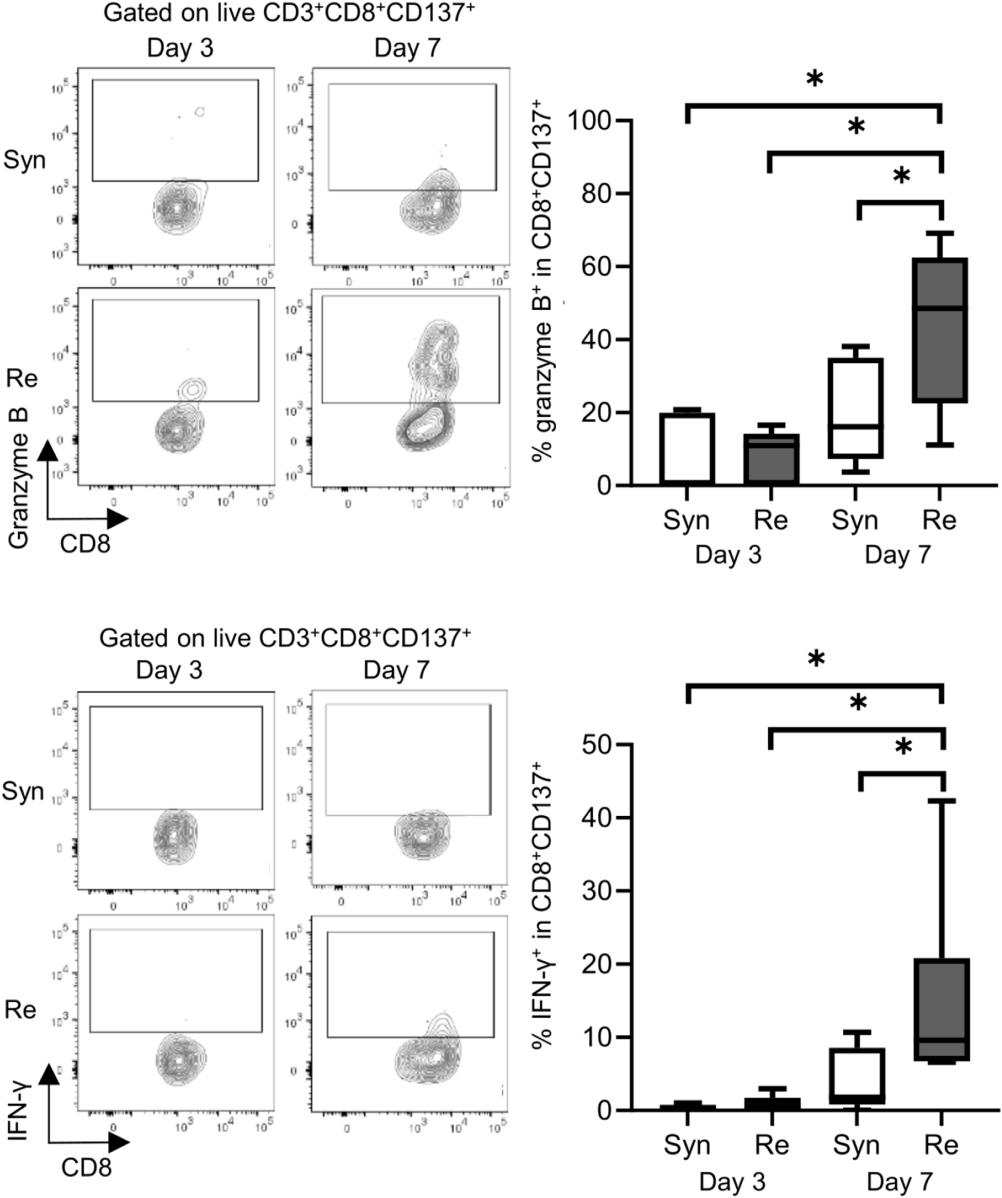
Functional analysis of donor-reactive T cells in the mouse skin transplant model. The representative flow plots show the expression of granzyme B (upper) and interferon gamma (IFN-γ) (lower) in CD137^+^ donor-reactive CD8^+^ T cells from recipients of the syngeneic model (Syn, C57BL/6 into C57BL/6) and rejection model (Re, BALB/c into C57BL/6). *p < 0.05. The data were generated from four independent experiments (n = 6). One-way ANOVA and Tukey’s multiple-comparison test were employed for statistical analysis.

**FIGURE 4:**
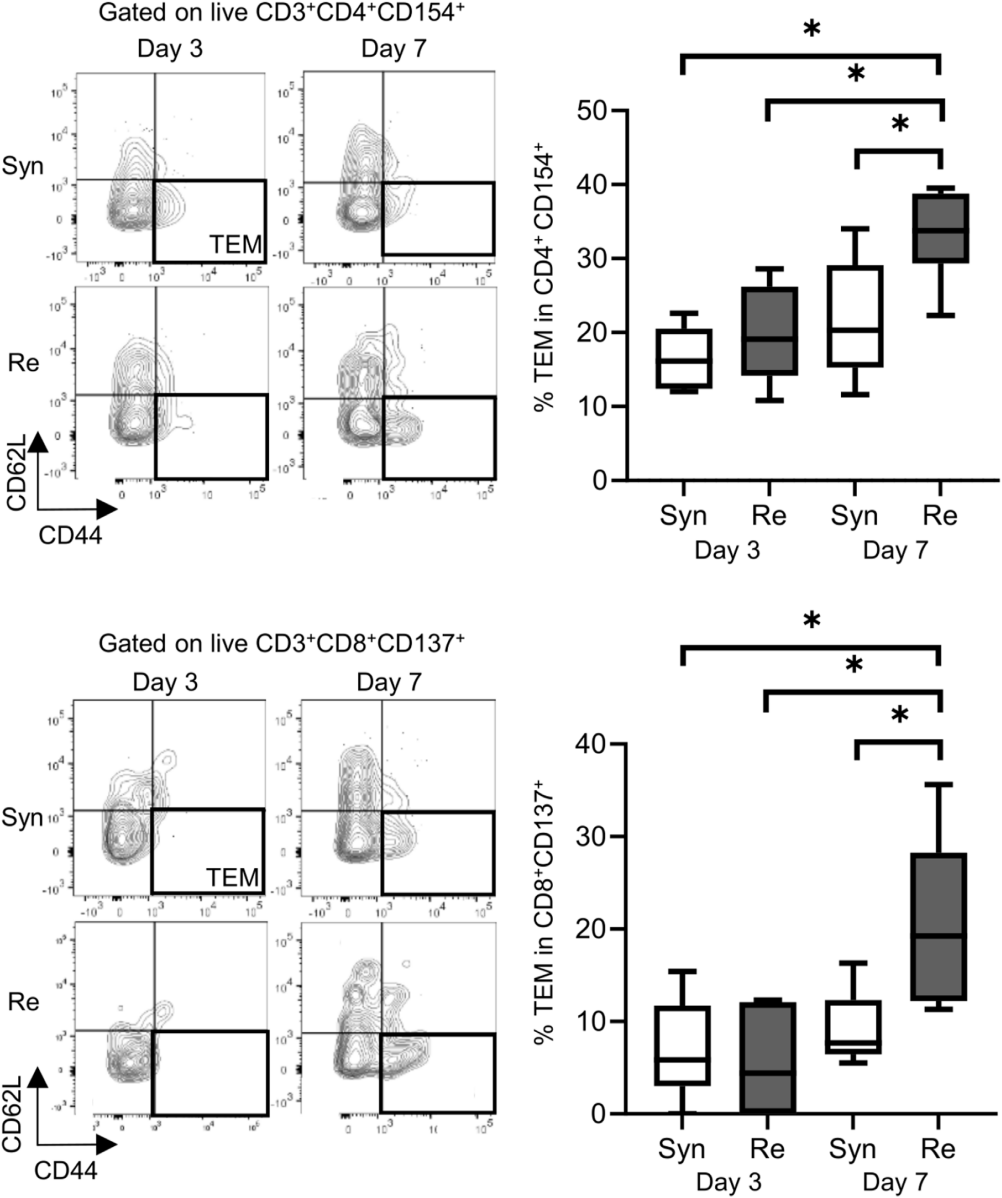
Proportions of effector memory T (TEM) cells (represented by CD44^+^ and CD62L^−^) in the mouse skin graft syngeneic model (Syn, C57BL/6 into C57BL/6) and rejection model (Re, BALB/c into C57BL/6). Proportions of TEM cells among CD154^+^ alloreactive CD4^+^ T cells (upper). Proportions of TEM cells among CD137^+^ alloreactive CD8^+^ T cells (lower). *p < 0.05. The data were generated from four independent experiments (n = 6). One-way ANOVA and Tukey’s multiple-comparison test were employed for statistical analysis.

Skin allografts were rejected from 7 to 15 days in the full MHC mismatched rejection model (BALB/c into C57BL/6) (MST 11 days, Supplementary Figure S1). We did not observe a sensitized reaction in peripheral CD4^+^ and CD8^+^ T cells at 3 days after transplantation, as determined using a proliferation assay (syngeneic vs. rejection model, median MI; CD4^+^ 0.18 vs. 0.08, p = 0.53 (upper) and CD8^+^ 0.20 vs. 0.10, p = 0.80 (lower), Figure 1). Seven days after transplantation, we observed a higher proliferation response of both CD4^+^/CD8^+^ T cells in the rejection model than in the syngeneic model (median MI; CD4^+^ 0.18 vs. 0.46, p < 0.05 (upper) and CD8^+^ 0.57 vs. 1.28, p < 0.05 (lower), Figure 1). The cATD assay revealed a sensitized immune response after skin transplantation at the same time point as the proliferation assay, showing a higher proportion of donor-reactive CD4^+^CD154^+^/CD8^+^CD137^+^ T cells than that in the syngeneic model at 7 days after transplantation (syngeneic vs. rejection model, CD4^+^CD154^+^ in total CD4^+^; 1.0% vs. 1.4%, p < 0.05 (upper) and CD8^+^CD137^+^ in total CD8^+^; 0.27% vs. 0.53%, p < 0.05 (lower), Figure 2). Donor-reactive T cells identified in this assay showed an increase in proportion and enhancement in function under antigen-specific stimulation in recipients sensitized with BALB/c mouse graft (Supplementary Figure S2). The multiparametric flowcytometric analyses demonstrated a unique functionality of donor-reactive CD8^+^ T cells in the rejection model; for instance, the production ability of the crucial effectors, GZMB and IFN-γ, was specifically enhanced in donor-reactive CD8^+^ T cells in the rejection model at 7 days after transplantation (syngeneic vs. rejection model, % positive for GZMB 16.1% vs. 54.7%, p < 0.05 (upper), and IFN-γ 1.82% vs. 8.18%, p < 0.05 (lower), Figure 3). As a proof of sensitization, effector memory T (TEM; CD44^+^CD62L^−^) cells were enriched in the donor-reactive population after transplantation (syngeneic vs. rejection model, median % TEM in CD4^+^CD154^+^ 20.2% vs. 32.9%, p < 0.05 (upper), and CD8^+^CD137^+^ 9.2% vs. 19.5%, p < 0.05 (lower), Figure 4).

### Quantitative and Qualitative Analyses of Donor-Reactive T Cells for Monitoring Tolerance Induction in the Treated Mouse Skin Transplantation Model

**FIGURE 5:**
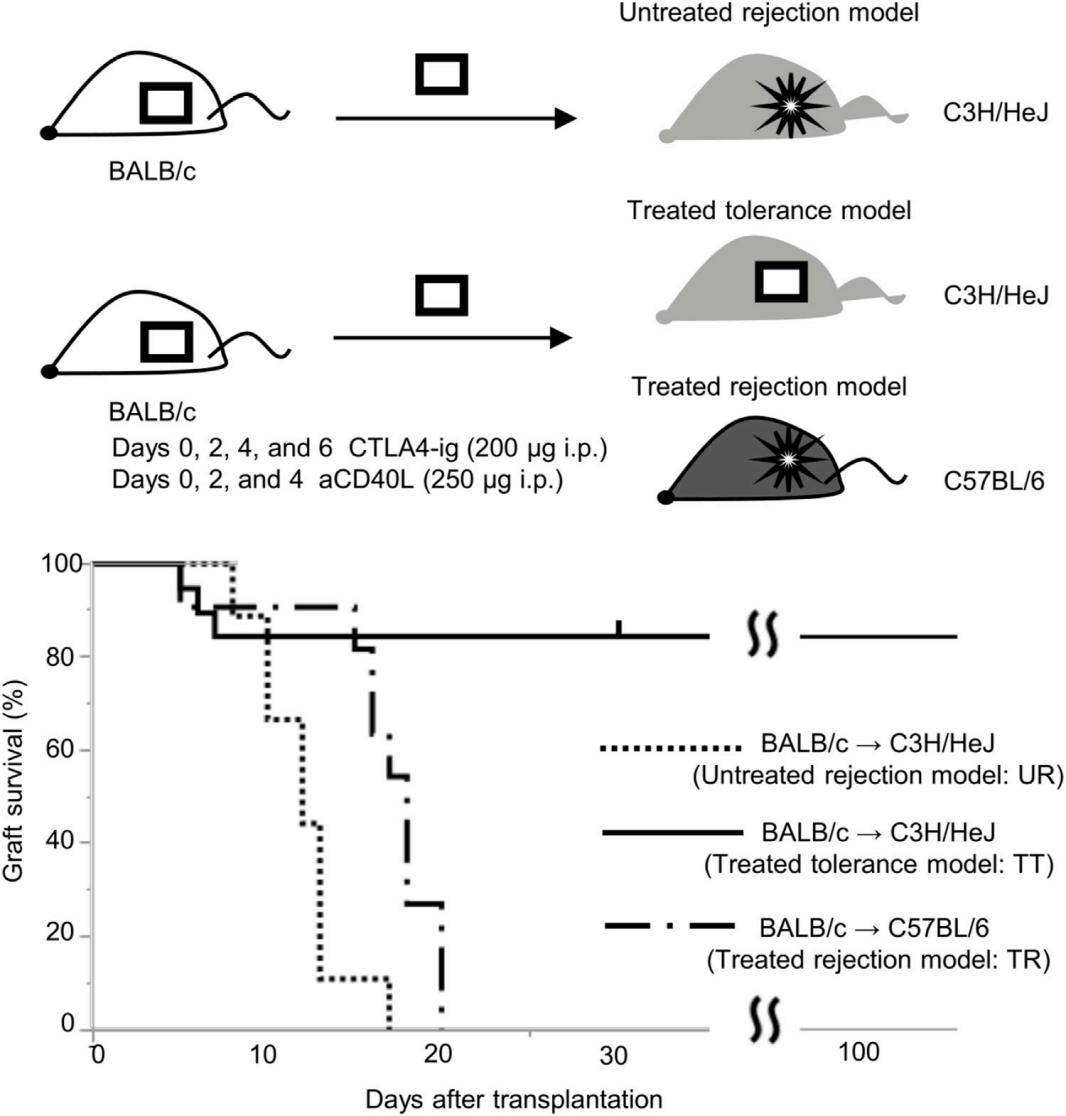
Graft survival curve of the skin transplantation mouse model with immunosuppression. Long-term engraftment was observed in C3H/HeJ recipients with BALB/c graft treated with CTLA-4 IgG and anti-CD154 antibody (treated tolerance model, TT, n = 19), whereas all BALB/c grafts were rejected in untreated C3H/HeJ recipients (untreated rejection model, UR, n = 9, MST: 12 days) and C57BL/6 recipients treated by tolerance induction (treated rejection model, TR, n = 12, MST: 18 days).

**FIGURE 6:**
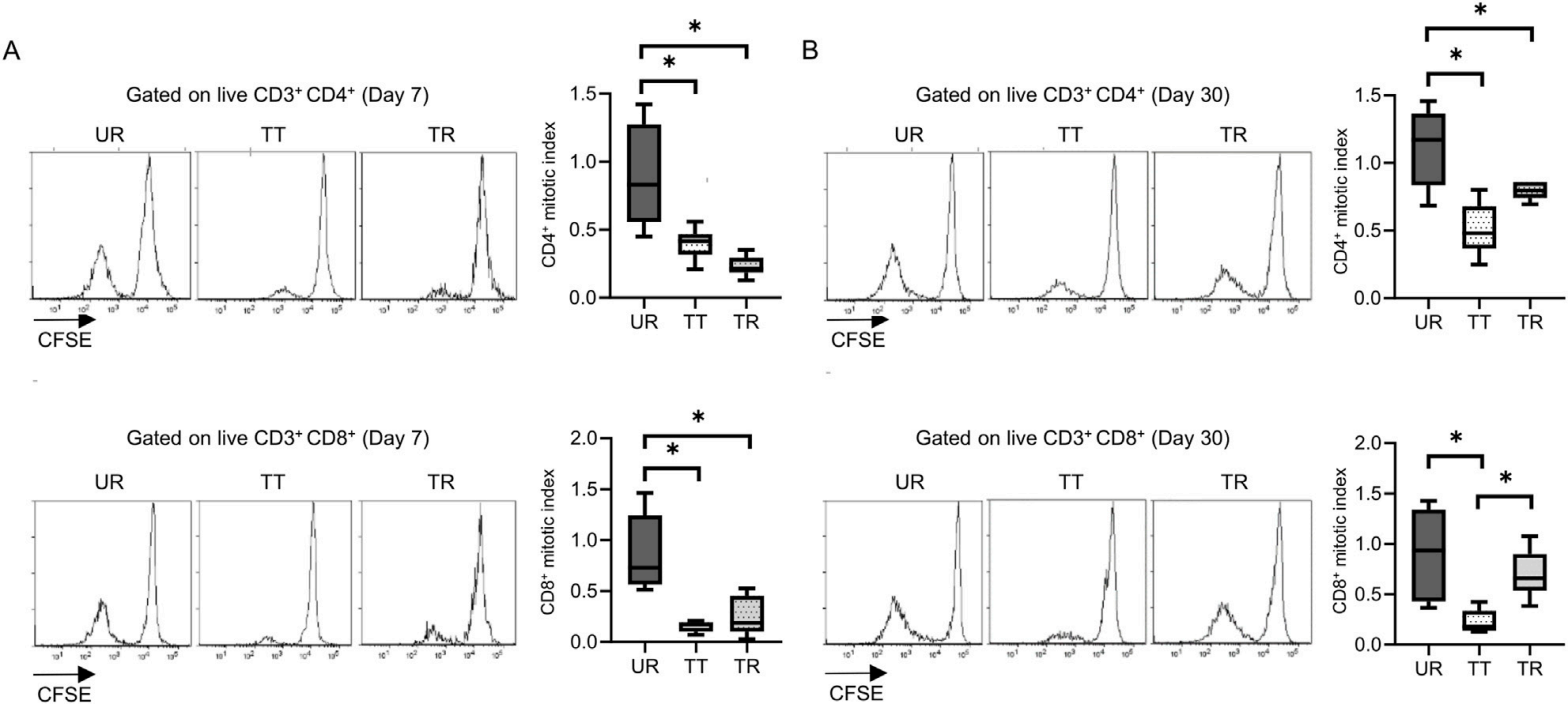
Proliferation assay after mouse skin transplantation with immunosuppression. Representative flow plots and box-and-whisker plots of the mitotic index show the proliferation capacity of CD4 and CD8 T cells from the untreated rejection model (UR, BALB/c into C3H/HeJ) and model treated with CTLA-4 IgG and anti-CD154 antibody (TT, BALB/c into C3H/HeJ or TR, BALB/c into C57BL/6) at **(A)** 7 and **(B)** 30 days after transplantation. *p < 0.05. The data were generated from three independent experiments (n = 6). One-way ANOVA and Tukey’s multiple-comparison test were employed for statistical analysis.

**FIGURE 7:**
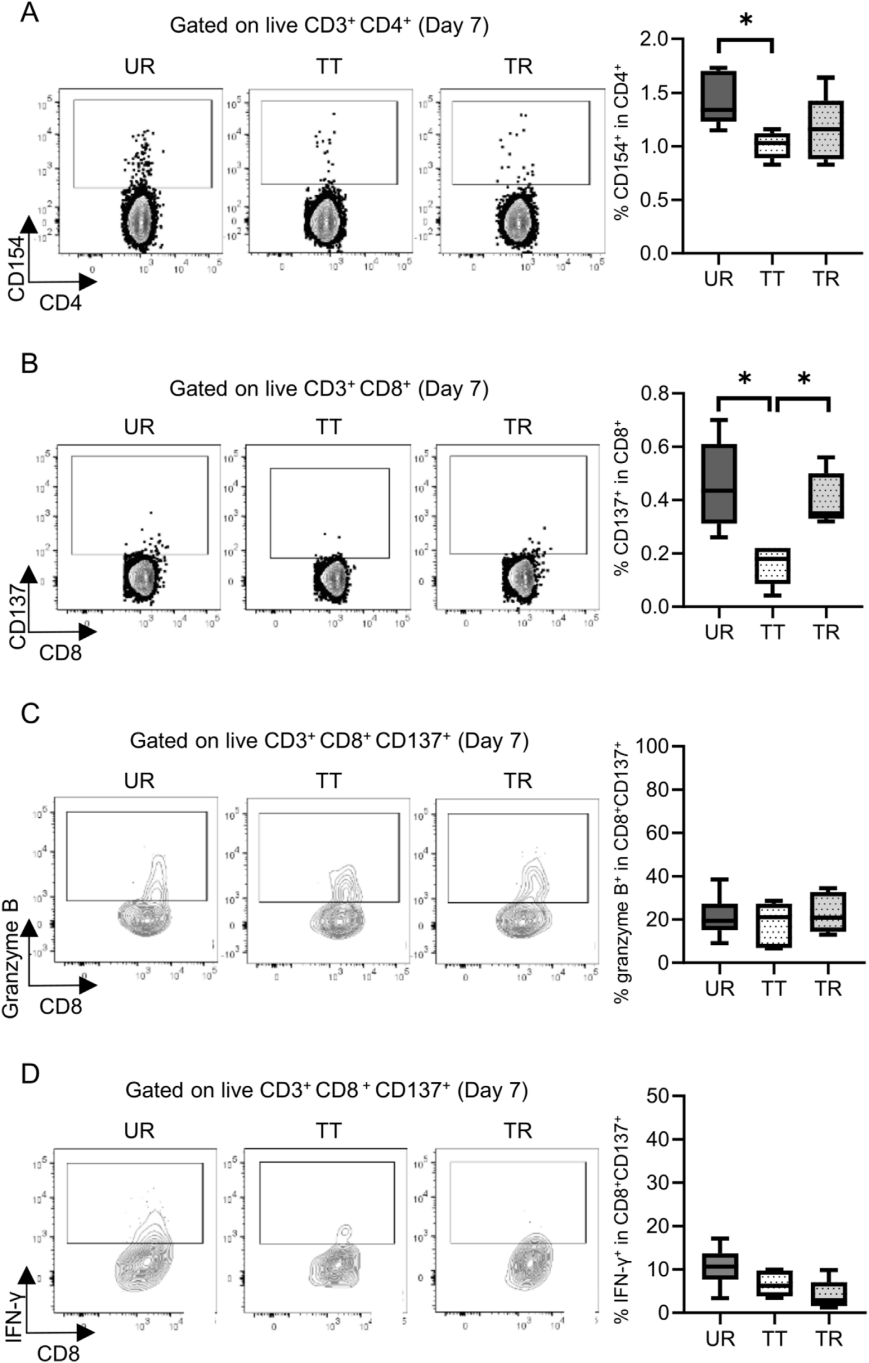
Detection and functional analysis of donor-reactive T cells using an alloreactive T-cell detection assay in the mouse skin transplant model at 7 days after transplantation. Representative flow plots show the alloreactive population defined by **(A)** CD154^+^ in CD4^+^ T cells and **(B)** CD137^+^ in CD8^+^ T cells and the expression of **(C)** granzyme B and **(D)** interferon gamma (IFN-γ) in CD137^+^ donor-reactive CD8^+^ T cells from the untreated rejection model (UR, BALB/c into C3H/HeJ) and model treated with CTLA-4 IgG and anti-CD154 antibody (TT, BALB/c into C3H/HeJ or TR, BALB/c into C57BL/6). *p < 0.05. The data were generated from three independent experiments (n = 6). One-way ANOVA and Tukey’s multiple-comparison test were employed for statistical analysis.

**FIGURE 8:**
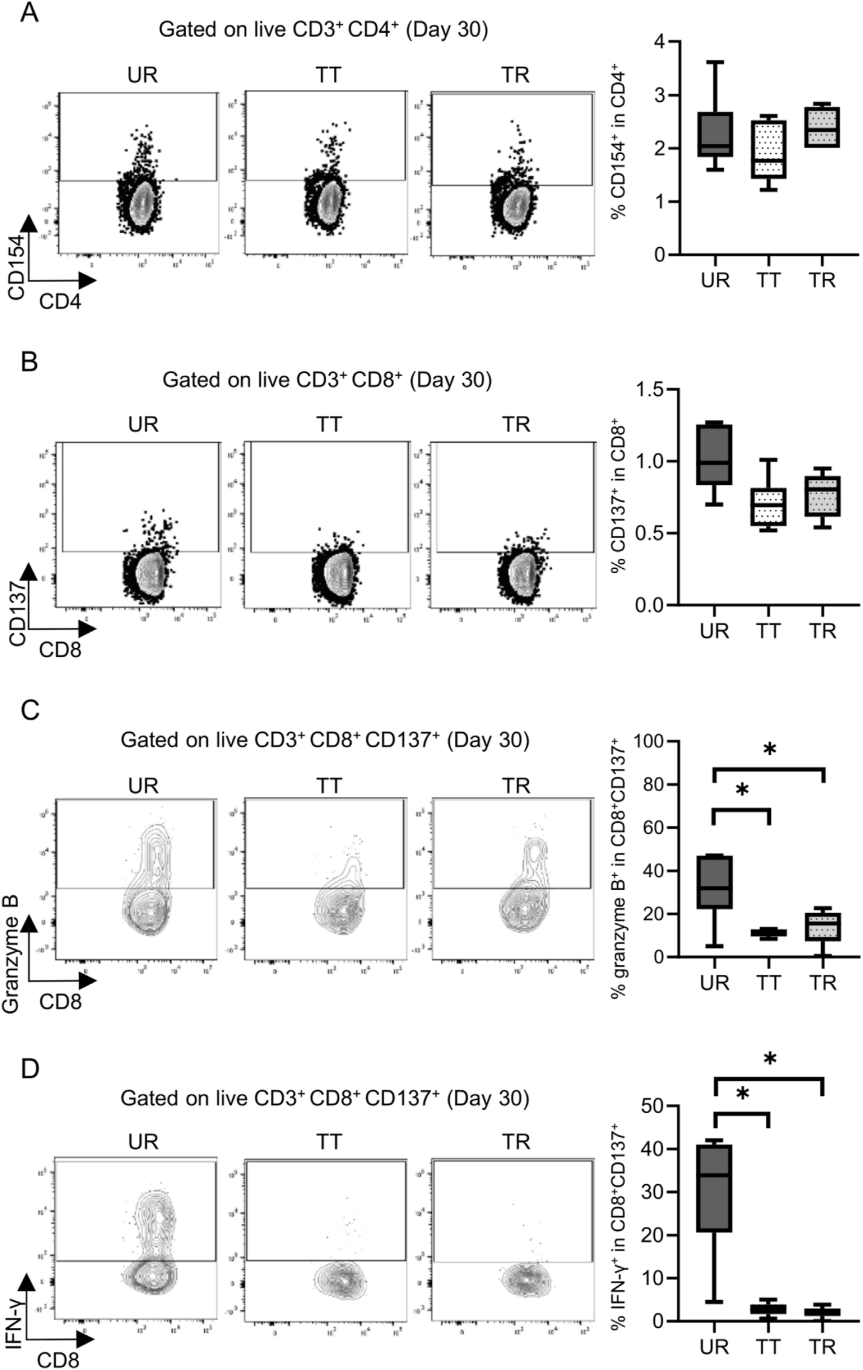
Detection and functional analysis of donor-reactive T cells using an alloreactive T-cell detection assay in the mouse skin transplant model at 30 days after transplantation. Representative flow plots show the alloreactive population defined by **(A)** CD154^+^ in CD4^+^ T cells and **(B)** CD137^+^ in CD8^+^ T cells and the expression of **(C)** granzyme B and **(D)** interferon gamma (IFN-γ) in CD137^+^ donor-reactive CD8^+^ T cells from the untreated rejection model (UR, BALB/c into C3H/HeJ) and model treated with CTLA-4 IgG and anti-CD154 antibody (TT, BALB/c into C3H/HeJ or TR, BALB/c into C57BL/6). *p < 0.05. The data were generated from three independent experiments (n = 6). One-way ANOVA and Tukey’s multiple-comparison test were employed for statistical analysis.

Permanent engraftment was observed in C3H/HeJ recipients of the full MHC mismatched BALB/c graft treated with CTLA-4 IgG and anti-CD154 antibody (treated tolerance (TT) model, ≥30-day survival was recorded in 16/19 animals, 84.2%), whereas all C57BL/6 recipients with the same immunosuppression eventually experienced allograft rejection within 20 days (treated rejection (TR) model, Figure 5). We investigated the immunological status at 7 and 30 days after transplantation, that is, before and after rejection, respectively. The proportion of FOXP3^+^ Tregs in CD4^+^ T cells was comparable, despite tolerance induction, between 7 and 30 days after transplantation (Supplementary Figure S3). The proliferation assay conducted at 7 days after transplantation showed a significant reduction in response to immunosuppression in both C3H/HeJ and C57BL/6 recipients compared with that in an untreated rejection (UR) model. However, the conventional proliferation readout results did not show the differential immune response at 7 days after transplantation between C3H/HeJ and C57BL/6 recipients, despite a different outcome (TT model vs. TR model, median MI; CD4^+^ 0.41 vs. 0.21, p = 0.44 and CD8^+^ 0.15 vs. 0.19, p = 0.70, respectively, Figure 6A). The cATD assay revealed that the proportion of CD8^+^ donor-reactive T cells in the TT model was lower than that in the TR model at 7 days after transplantation (TT model vs. TR model, median % donor-reactive CD8^+^; 0.18% vs. 0.35%, p < 0.05, Figures 7A, B). The GZMB- and IFN-γ-producing capacity of the CD8^+^ donor-reactive T cells was comparatively low in the three groups (Figures 7C, D). Regardless of the final outcome, models with immunosuppression exhibited impaired memory formation in donor-reactive T cells (Supplementary Figure S4). At 30 days after transplantation, the proliferation assay showed a lower MI of CD8^+^ T cells for the response of the TT model than that for the response of both UR and TR models (TT model vs. UR and TR models, median CD8^+^ MI: 0.18 vs. 0.93 and 0.66, p < 0.05, respectively, Figure 6B). The cATD assay performed at 30 days after transplantation revealed that donor-reactive T cells were detectable in the TT model, similar to those in the UR and TR models (UR vs. TT vs. TR, %CD4^+^CD154^+^ in total CD4^+^ was 2.01%, 1.77%, and 2.35%, %CD8^+^CD137^+^ in total CD8^+^ was 1.00%, 0.7%, and 0.81%, respectively, Figures 8A, B). As expected, the functionality of donor-reactive CD8^+^ T cells in the TT model was lower than that in the UR model (UR vs. TT model, % positive in donor-reactive CD8^+^ T cells, GZMB; 31.9% vs. 11.2%, p < 0.05, and IFN-γ; 33.9% vs. 3.04%, p < 0.05, respectively, Figures 8C, D). However, there were no differences in functionality and memory formation between the TT and TR models (Supplementary Figures S4, S5).

## Discussion

Allogeneic reactive T cells play a pivotal role in the process of promoting or conversely regulating rejection in allogeneic solid organ transplantation [1]. Understanding the characteristics and behavior of alloreactive T cells is vital for assessing the immune response after allogeneic transplantation [4]. MLR is a classical but practical method to assess allo-response. The precursor frequency of alloreactive T cells has been reported to be 1%–10% under various assay conditions and readouts in both murine and human T-cell repertoires [14–17]. Proliferation, which requires a culture period of 4–5 days, has been widely used as an accessible readout to visualize and quantify the responsiveness of alloreactive T cells using MLR. However, with advancements in flow cytometry technology, it has become feasible to perform multiparametric evaluations of rare populations of less than 1%. This finding suggests the possibility of assessing these infrequent alloreactive T cells without the need for proliferation. In line with this prospect, a previous study demonstrated that the cATD assay, using activated allogeneic B-cell stimulators and very early activation markers, enables the detection of alloreactive T cells with high precision in a short-term culture system [8]. In the present study, we validated the utility of the cATD assay for rapid evaluation of donor-reactive T cells in an *in vivo* transplantation model. The usefulness of CD154 and CD137 for detecting antigen-specific CD4^+^ and CD8^+^ T cells as rapid-activating molecules has been demonstrated using viral peptides and toxins, respectively [18–20]. CD154 is preferentially expressed on effector CD4^+^ T cells and memory CD8^+^ T cells [21]. Although CD137 expression can be induced on CD4^+^ T cells, the combination of CD137^+^CD154^−^ expression after allo-stimulation has been reported to delineate activated FOXP3^+^ regulatory T cells that exhibit a specific suppressive capacity against corresponding allo-stimulation [22, 23]. Single-cell TCR analysis has revealed that CD137 expression on CD8^+^ T cells after allogeneic stimulation is a marker for oligoclonal expanded alloreactive T cells during acute cellular rejection (ACR) after lung transplantation [24]. Moreover, alloreactive CD154 expression on CD8^+^ memory T cells has been reported to be associated with acute rejection after pediatric liver, intestine, and kidney transplantation [25–27]. Although CD154 could be used as a candidate for predicting rejection by analyzing memory CD8^+^ T cells, CD137 can be used as a marker to detect a variety of CD8^+^ T-cell subsets including a substantial portion of naïve populations [20]. Consistent with the results of the previous study, we observed a considerable proportion of a naïve phenotype in donor-reactive CD8^+^ T cells using CD137 detection. CD137 alloreactive CD8^+^ T cells showed greater functional molecule expression than those detected by CD154 in our rejection model mice (Supplementary Figure S6).

In clinical settings, the cATD assay enables repeated monitoring of circulating alloreactive T cells. The significance of alloreactive T-cell clones in circulation as the pathological effector of rejection after transplantation may be controversial. A recent TCR repertoire analysis using next-generation sequencing revealed that expanded circulating T-cell clones during ACR were observed in the circulation before ACR after lung [24], liver [28], and kidney transplantation [29, 30]. Furthermore, expanded clones in circulation have been reported to overlap with infiltrated T-cell clones in the liver [28] and kidney allografts [29, 30]. An interesting case report of malignant melanoma treated with an immune checkpoint inhibitor after kidney transplantation indicated that the alloreactive T-cell cluster in renal biopsy identified through single-cell RNA sequencing overlapped with circulating clones, which were identified both before and after rejection of the allograft [29]. According to these observations, we believe that circulating alloreactive T cells reflect immune responses after solid organ transplantation.

In the current era where organ transplantation is a standard therapy for patients with organ failure, a standard approach to monitor harmful alloimmune responses is lacking [31]. A previous study reported the usefulness of quantified proliferation in MLR to diagnose immunological rejection [32]. The proliferation and cATD assays assess different time points and readouts, suggesting that they can identify different T-cell populations. During the proliferation assay, *in vitro* culture of T cells is performed over several days to amplify them and obtain T cells of various developmental stages. On the contrary, the cATD assay detects the population that responds rapidly in MLR initiated through overnight culturing, which may indicate a highly primed status and is directly linked to impending rejection. As this assay assesses alloreactivity through a direct pathway, missing the component through indirect pathways could be a limitation when monitoring long-term allo-response after transplantation. However, we believe that its relevance to *in vivo* acute rejection models makes it a useful tool for immune monitoring.

We observed different outcomes and immunological findings in tolerance induction between C3H/HeJ (TT) and C57BL/6 (TR) recipients. C3H/HeJ mice express a dysfunctional toll-like receptor 4, which reduces macrophage and B-cell proliferation and antigen-presenting capabilities, possibly leading to different immune responses and outcomes [33]. Interestingly, the cATD assay showed quantitatively different priming status of donor-reactive CD8^+^ T cells between the TT and TR models before rejection. After rejection when the rejected graft was lost, the cATD assay did not show differential findings between the TT and TR models; however, the proliferation assay reliably showed sensitization potential in the TR model, based on the results obtained 30 days after transplantation. These findings may be attributed to the feature of alloreactive T cells detected using the cATD assay. This study has some limitations. Notably, the immunological response in skin transplantation is potentially different from that in organ transplantation. Investigation of other organ transplant models and clinical samples could further validate the relevance of the findings of the present study across diverse transplantation settings. However, the cATD assay, which enables real-time and repeatable detection of donor-reactive effectors, might be clinically relevant in diagnosing harmful allo-responses directly linked to the region responsible for rejection. Future research should compare the TCR repertoire of reactive T cells at rejection or upon achieving tolerance between proliferation and cATD assays to obtain differential immunological information. Multifaceted evaluation through the cATD assay facilitates the investigation of superior functional molecules and biomarkers for monitoring clinical conditions such as tolerance status. Additionally, it enables the retrieval of rare live alloreactive T-cell populations for downstream investigation via fluorescence-activated cell sorting and provides valuable information for further studies in the field of translational research.

In conclusion, the cATD assay using CD154 and CD137 as alloreactive markers effectively distinguished immune responses in *in vivo* mouse transplantation models, highlighting its potential to facilitate prompt quantitative and qualitative estimation of alloreactive T cells after allogeneic transplantation.

## Data Availability

The original contributions presented in the study are included in the article/Supplementary Material, further inquiries can be directed to the corresponding author.

## Abbreviations

APC, antigen-presenting cell; cATD, comprehensive alloreactive T-cell detection; CFSE, carboxy fluorescein succinimidyl ester; GZMB, granzyme B; IFN-γ, interferon gamma; mAb, monoclonal antibody; MHC, major histocompatibility complex; MLR, mixed lymphocyte reaction; TCR, T-cell receptor.
