# Supplementary figures and images for: Discrimination of Anti-Donor Response in Allogeneic Transplantation Using an Alloreactive T-Cell Detection Assay
Source: Transpl Int. 2025 Jan 28;38:13879. doi: 10.3389/ti.2025.13879 (PMC11810569; doi:10.3389/ti.2025.13879)

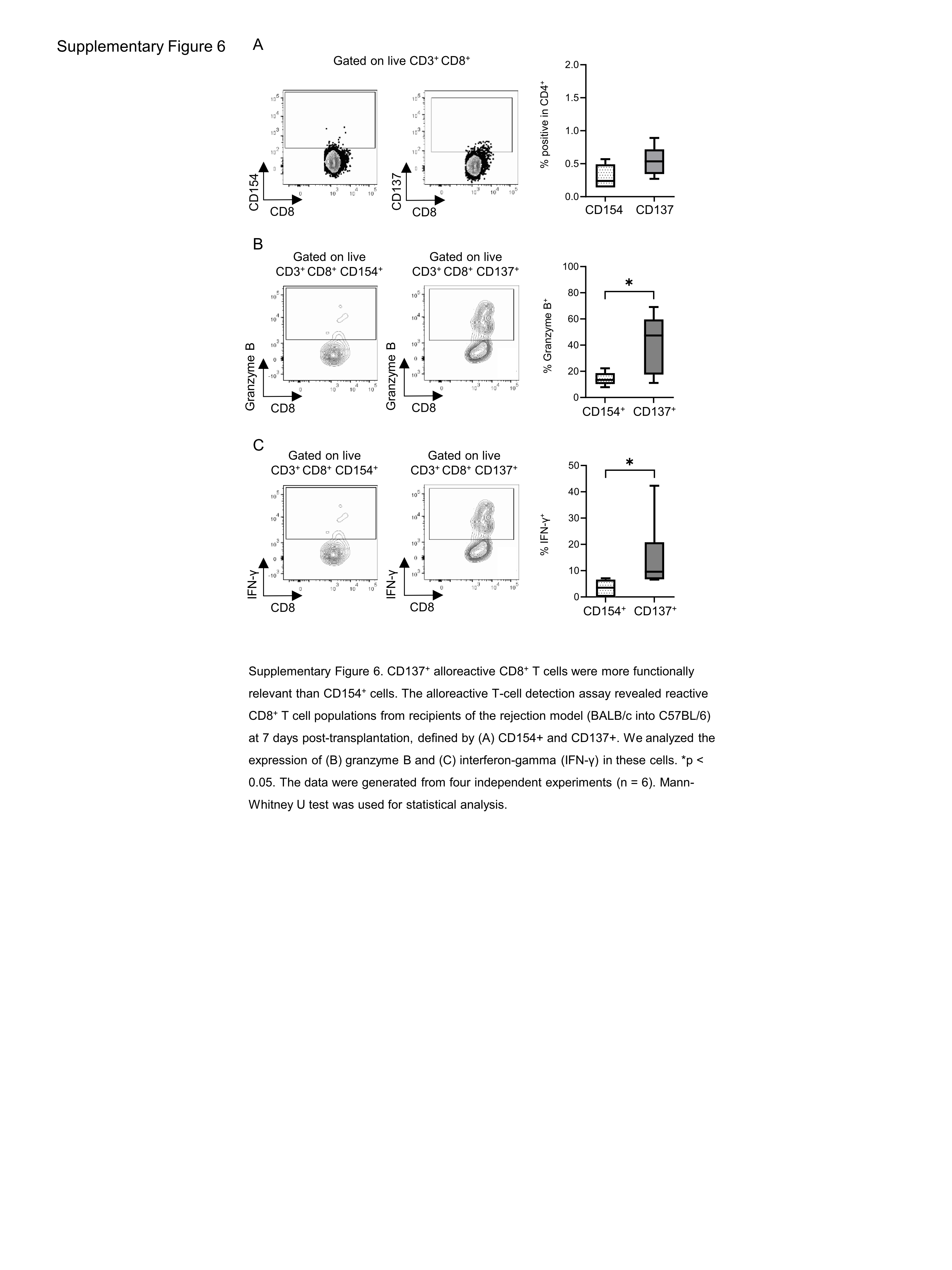

Supplement: Supplementary file 1 [file Image6.TIF]

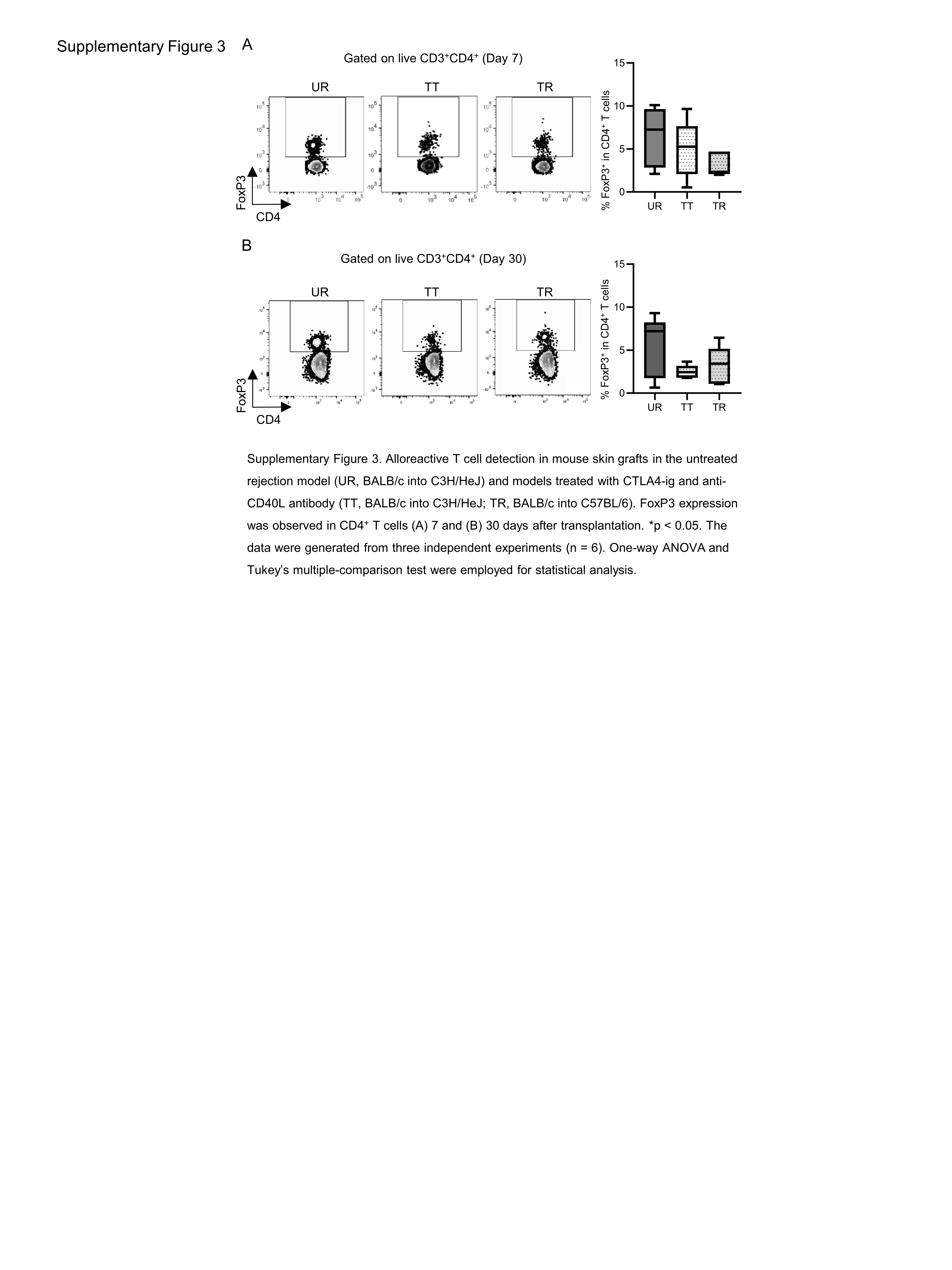

Supplement: Supplementary file 2 [file Image3.TIF]

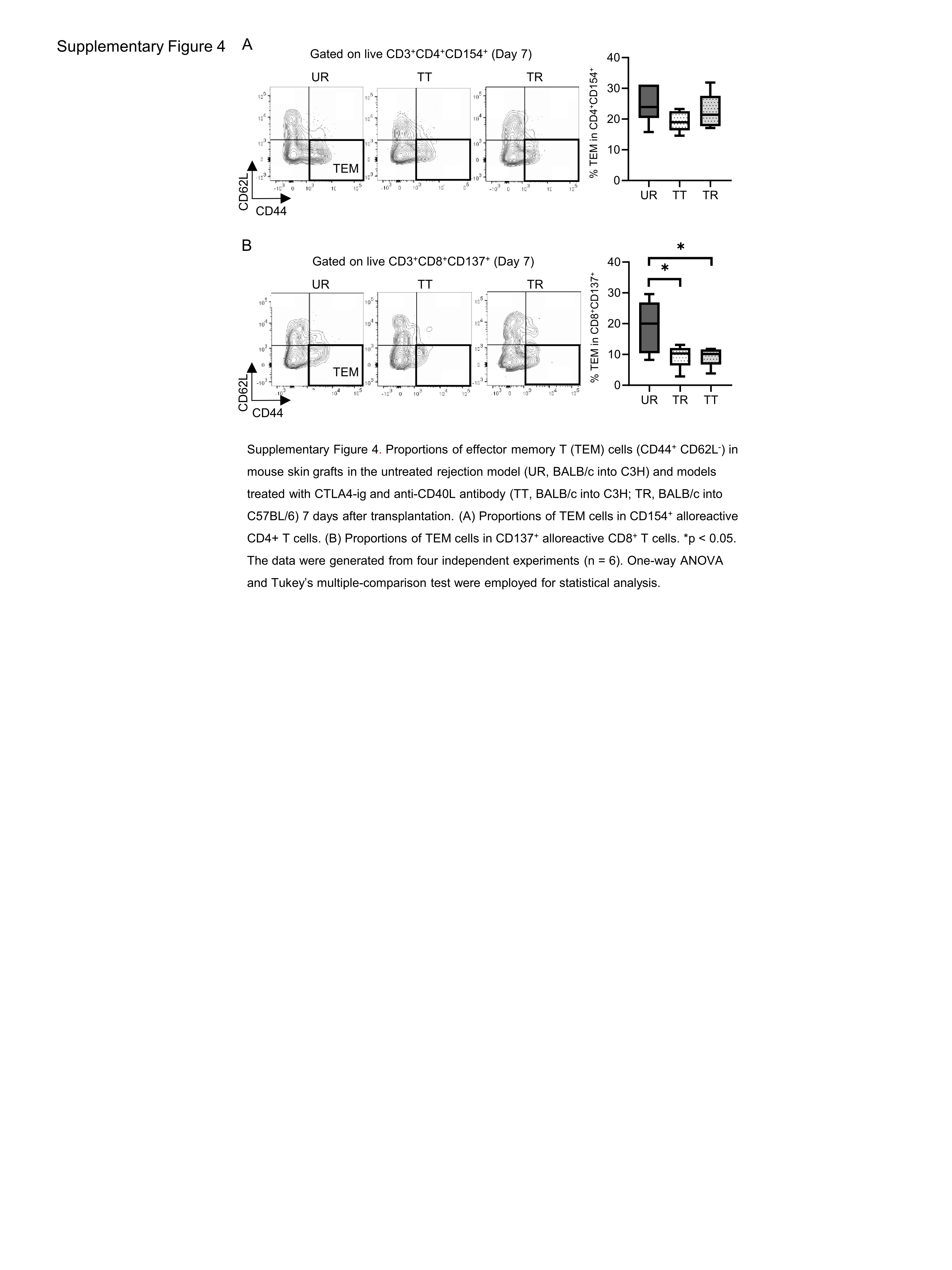

Supplement: Supplementary file 3 [file Image4.TIF]

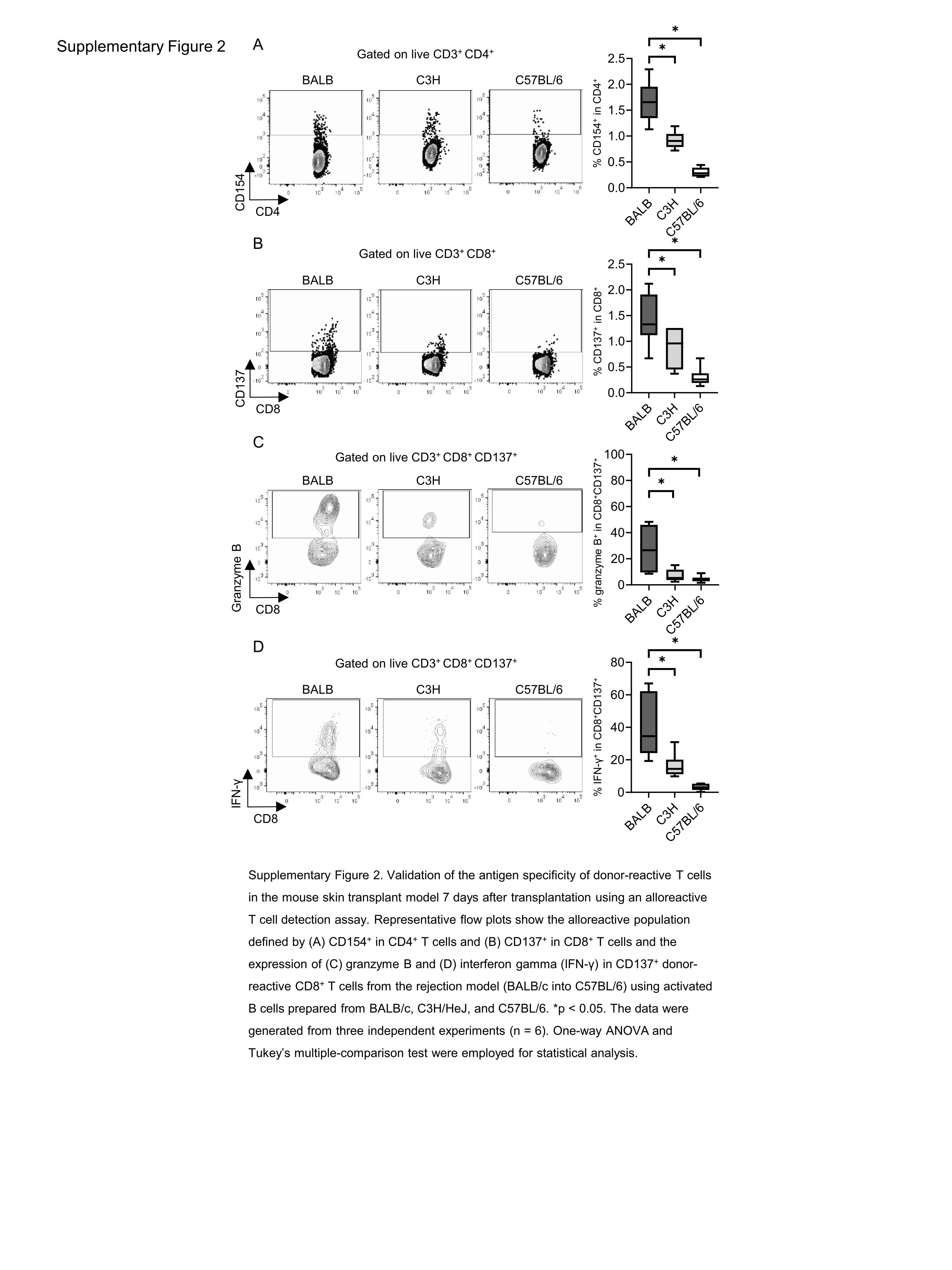

Supplement: Supplementary file 4 [file Image2.TIF]

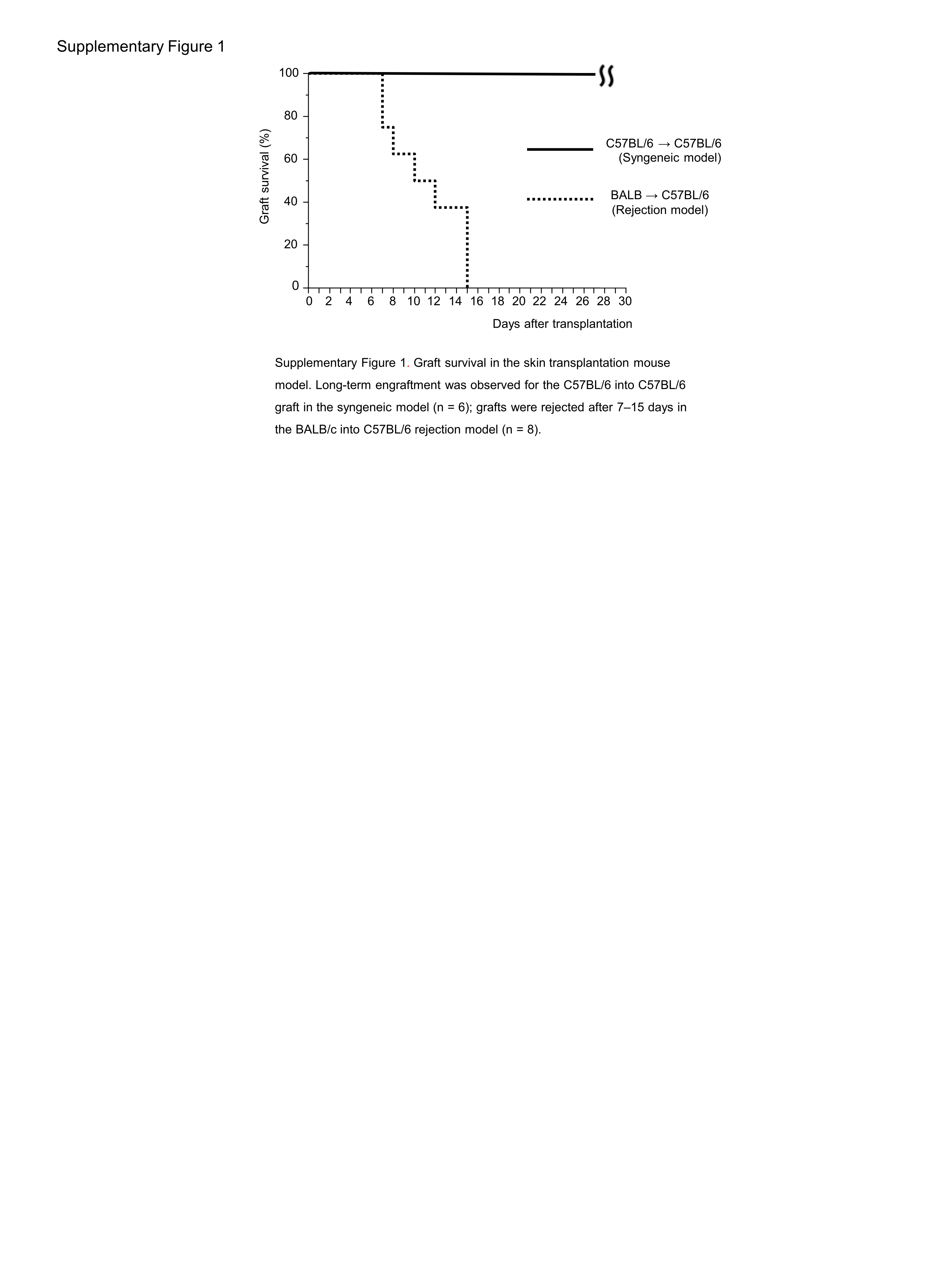

Supplement: Supplementary file 5 [file Image1.TIF]

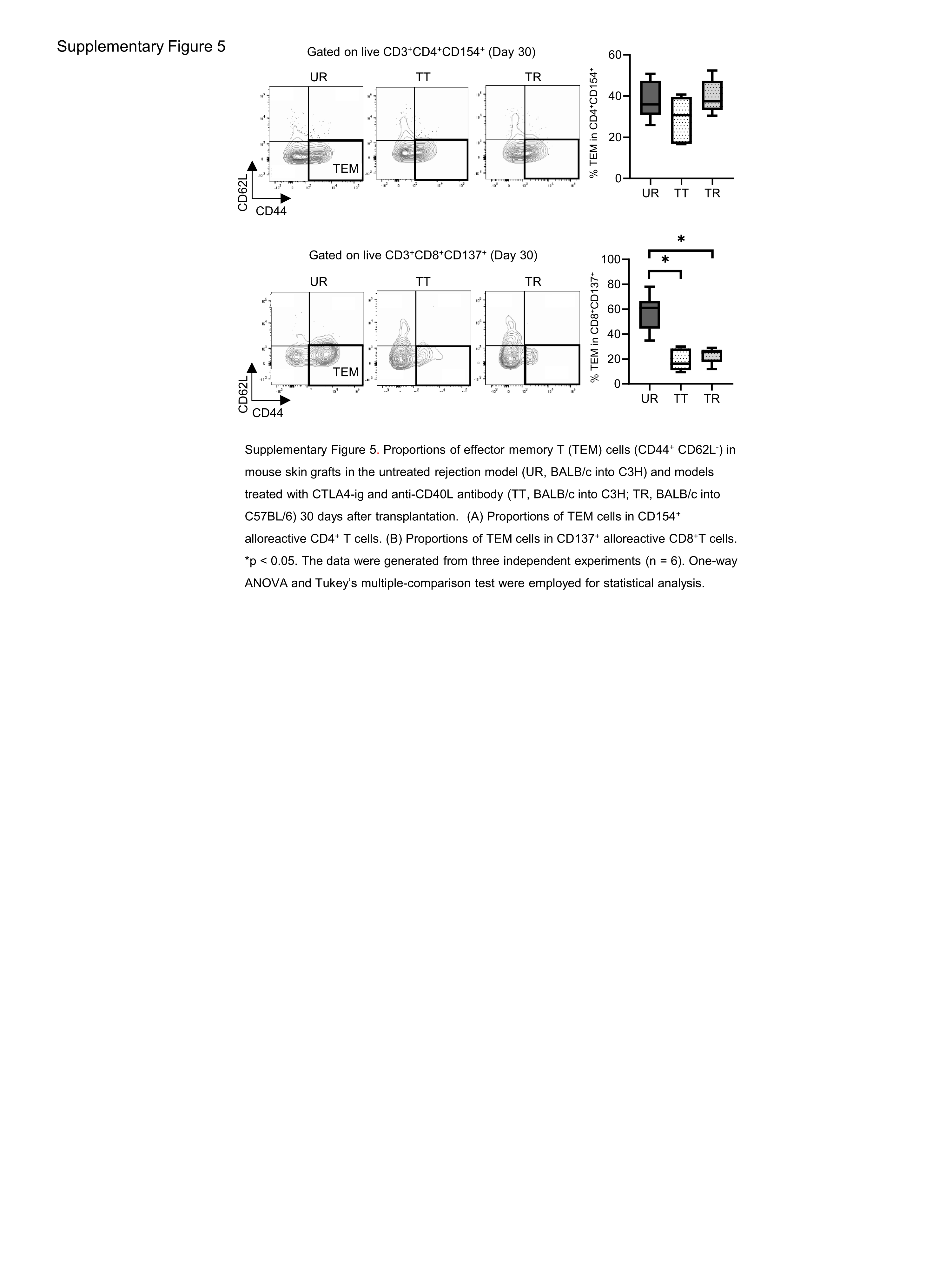

Supplement: Supplementary file 6 [file Image5.TIF]
